# Efficient dual-negative selection for bacterial genome editing

**DOI:** 10.1186/s12866-020-01819-2

**Published:** 2020-05-24

**Authors:** Francesca Romana Cianfanelli, Olivier Cunrath, Dirk Bumann

**Affiliations:** Biozentrum, University of Basel, CH-4056 Basel, Switzerland

**Keywords:** *Salmonella*, Homologous recombination, Mutagenesis, Gene manipulation, MDR

## Abstract

**Background:**

Gene editing is key for elucidating gene function. Traditional methods, such as consecutive single-crossovers, have been widely used to modify bacterial genomes. However, cumbersome cloning and limited efficiency of negative selection often make this method slower than other methods such as recombineering.

**Results:**

Here, we established a time-effective variant of consecutive single-crossovers. This method exploits rapid plasmid construction using Gibson assembly, a convenient *E. coli* donor strain, and efficient dual-negative selection for improved suicide vector resolution. We used this method to generate *in-frame* deletions, insertions and point mutations in *Salmonella enterica* with limited hands-on time. Adapted versions enabled efficient gene editing also in *Pseudomonas aeruginosa* and multi-drug resistant (MDR) *Escherichia coli* clinical isolates.

**Conclusions:**

Our method is time-effective and allows facile manipulation of multiple bacterial species including MDR clinical isolates. We anticipate that this method might be broadly applicable to additional bacterial species, including those for which recombineering has been difficult to implement.

## Background

Genetic engineering is fundamental for molecular analysis of genotype-phenotype relationships, and for determining the function of previously uncharacterized genes [1–3]. Site-specific mutagenesis can be achieved using different methods. Traditionally, marker-free genetic manipulations were obtained using consecutive single-crossovers mediated by endogenous recombinases [4, 5]. A suicide vector is first integrated in the desired location using homologous recombination. Bacteria, in which a subsequent second crossover results in loss of the integrated plasmid, can then be selected using counter-selection markers [6–9]. However, counter-selection is often suboptimal resulting in a need to screen many clones for the desired event [10, 11]. Later, the λ-Red recombineering technology, a phage-based homologous recombination system based on linear DNA transfer and an exogenous recombinase, was introduced [8, 12–15]. Scarless mutations can be obtained when combining this method with a counter-selection marker [16–19]. Currently, λ-Red recombineering is the method of choice for introducing genetic manipulations in *S. enterica* and *E. coli* [20] but it has been difficult to implement in several other bacterial species such as *Pseudomonas aeruginosa*. Recently, clustered regularly interspaced short palindromic repeats (CRISPR)-Cas has revolutionized eukaryotic genome editing [21–23], but this strategy is more cumbersome for bacteria with limited recombination activities [24–26].

**Fig. 1:**
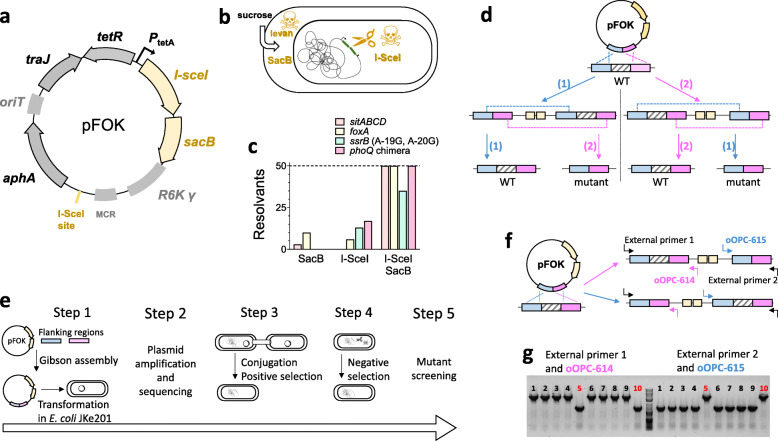
An optimized method for genome editing in *Salmonella enterica*. **a** Map of suicide plasmid pFOK (*aphA*, aminoglycoside phosphotransferase gene conferring resistance to kanamycin*; I-sceI* gene encoding meganuclease; *oriT*, origin of conjugational transfer; *P*_tetA_, *tetA* promoter; R6K γ *ori*, pi-dependent origin of replication; *sacB*, levansucrase gene; *tetR*, tetracycline repressor gene; *traJ*, transcriptional activator for conjugational transfer genes; MCR, multi cloning region containing EcoRI and BamHI recognition sites). **b** Mechanisms of negative selection for SacB and I-SceI, **c** Efficiency of negative selection for various chromosomal loci (*sitABCD* deletion - orange, *foxA* deletion - yellow, *ssrB* point mutation – green, and *phoQ* chimeric insertion - magenta [30]) using either SacB or I-SceI, or a combination of both. Fifty colonies were screened for each mutation. **d** Schematic representation of the consecutive single crossover procedure. Recombination can occur in one of the two homologous sequences (routes 1 and 2). Only alternate single crossover events involving both homologous sequences lead to the desired mutation, while two consecutive single crossovers in the same regions lead to reversion to wild-type (WT) **e** Overview of the entire procedure. Ideally, each step can be completed in one working day. **f** Schematic representation of preferential recombination in the right flanking region. External primers 1 and 2 together with plasmid-specific primers oOPC-614 and oOPC-615 can be used to screen co-integrant clones to reveal such bias and to identify rare variants for promoting mutant generation in the second single crossover. **g** Recombination bias for *foxA* deletion. PCR results of ex-conjugant screening using external primer 1 (oOPC-396) / oOPC-614 or external primer 2 (oOPC-397) / oOPC-615. Rare ex-conjugants (clones 5, 10) with recombination in the non-preferred flanking region were used for subsequent counter-selection

Here, we combined several improvements for establishing a time-efficient versatile method for consecutive single cross-overs in multiple bacterial species. We used rapid Gibson assembly of PCR products [27] to generate suicide vectors with dual negative selection mediated by I-SceI and SacB [28, 29] (Fig. 1a), which increased counter-selection efficiency to 100% for nearly all tested deletions, insertions and point mutations. We employed an *E. coli* donor strain that simplifies donor removal after conjugation and avoids common problems with contaminating phages [31]. We used different positive selection markers that enable selection in many bacterial species, including MDR pathogens [32]. Combination of these elements yielded a reliable and fast method for genetic engineering of multiple bacterial species that, in concert with a simplified screening procedure, minimized hands-on time and significantly accelerated genome editing in our lab.

## Results

Our goal was a rapid and efficient genetic editing method with minimal hands-on time. For this purpose, we combined rapid plasmid construction using Gibson assembly [27], a phage-free, *pir-*carrying (for propagation of *R6Kγ* plasmids), diaminopimelic acid (DAP)-dependent *E. coli* donor strain JKe201 [31] for plasmid amplification and conjugation, with subsequent facile removal of donor in absence of DAP, and an improved dual-negative counter-selection. We generated suicide vectors from PCR fragments with automatically designed primers using Gibson assembly [27]. Each vector carries commonly used genetic elements for conditional propagation (“suicide vector” with pi-dependent replication from *R6Kγ*), conjugation (*oriT*, *traJ*) and selection for two sequential single-crossovers. For the first positive selection, we used *aphA* conferring resistance to kanamycin (pFOK, Fig. 1a).

A major limitation to efficient genetic editing using two consecutive single-crossovers has been inefficient counter-selection of the second recombination, in part due to inactivating mutations in the negative selection marker [33]. We tested counter-selection efficiency in multiple *Salmonella* loci using the commonly used markers *sacB* or *I-sceI* (Fig. 1b). *sacB* codes for levan sucrase, which confers sensitivity to sucrose because of accumulation of the toxic product levan in the periplasm [28]. *I-sceI* codes for the restriction enzyme I-SceI, which causes lethal DNA double-strand breaks when a I-SceI recognition sequence is present on the genome [29]. To assess counter-selection efficiency of SacB or I-SceI singly, we generated plasmid variants (pOPC-001 and pOPC-003) differing just in the counter-selection. Counter-selection was suboptimal for both markers with marker-free clones representing none or only a minority of the recovered colonies (Fig. 1c). Consequently, many colonies had to be tested for finding the desired clones. To overcome this problem, we generated a new suicide vector, pFOK, combining both *sacB* and *I-sceI* under the regulatory control of the TetR regulator (Fig. 1a). We tested the TetR system using the green fluorescent protein (GFP) as reporter on the same pSC101 backbone and found no detectable GFP fluorescence above the autofluorescence background in absence of the inducer anhydro-tetracycline indicating limited leakiness in our conditions (Supplementary Fig. S1). Cells carrying the conditional dual-negative selection cassette under control of the TetR system showed no decrease in cloning efficiency but efficient negative selection in presence of sucrose and anhydro-tetracycline, yielding only, or a large majority, of resolvants that had successfully cured pFOK from their chromosome (Fig. 1c). A similar dual-negative selection has been previously described for Gram-positive bacteria [34]. The ratios for the two alternative results – mutation or reversion back to wild-type – varied between the individual mutants (Supplementary Fig. S2).

**Fig. 2:**
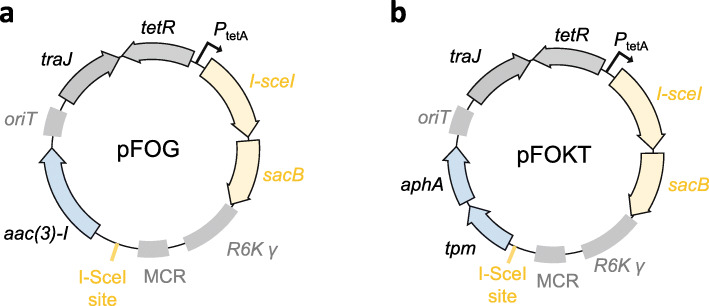
Maps of alternative suicide plasmids. **a** pFOG carrying *aac (3)-I* which confers resistance to gentamicin. **b** pFOKT carrying *tpm* coding for thiopurine-S-methyltranferase which confers resistance to tellurite

To expand our gene manipulation method to other bacterial species, including those for which λ-Red recombineering has not yet been established, we used alternative positive selection markers. This included *aac (3)-I*, coding for a aminoglycoside N-acetyltransferase that confers resistance to gentamicin which can be used as an alternative in bacteria, including *Pseudomonas aeruginosa*, which are resistant to kanamycin but susceptible to gentamicin (pFOG, Fig. 2a). We confirmed the utility of pFOG by deleting the *mexAB* operon in *P. aeruginosa* and observed 50 resolvants among 50 tested colonies (100%) after negative selection. As an alternative, we combined *aphA* with a second positive marker, *tpm*, yielding suicide vector pFOKT (Fig. 2b). *tpm* codes for a thiopurine-S-methyltranferase conferring resistance to tellurite [35]. This plasmid can be used for multi-drug resistant (MDR) bacteria for which the choice of positive selection markers is limited [32]. To limit toxic exposure to volatile dimethyl telluride, we used kanamycin for suicide vector generation and used tellurite only for the positive selection of ex-conjugants. We confirmed the utility of pFOKT by deleting *tolC* with high efficiency in a multi-drug resistant clinical *Escherichia coli* isolate [32] and again observed 50 resolvants among 50 tested colonies (100%) after negative selection.

In some cases, the second single-crossover had a high bias for resolution to wild-type loci (instead of the desired mutant). This was usually due to differences in recombination frequency between the two flanking regions. PCR primers (oOPC-614 and oOPC-615) that bind in the plasmid, combined with chromosomal primers outside the flanking regions in the merodiploids (Fig. 1f, g), enabled detection of such biases. For these cases, we selected ex-conjugants in which the first single-crossover had occurred in the non-preferred flanking region. In these clones, we often observed frequent resolution to mutant loci during the second single-crossover (deletion of *foxA*, Supplementary Fig. S2).

Altogether, the whole protocol from initial plasmid construction to scar-less sequence-verified mutant strains (Fig. 1e) was completed within five working days with minimal hands-on time for 23 of 30 *Salmonella* mutants. We obtained all residual mutants after additional optimization of the initial PCR (four mutants), prolonged cultivation times for mutants with reduced growth (two mutants), or screening for biased recombination as shown in Fig. 1f, g (one mutant – Δ*foxA*). The *Escherichia tolC* mutant was also completed within 5 working days, while slow growth of *Pseudomonas* at 28 °C during the negative selection required a total of 6 working days. In all cases, we obtained 100% resolvants after a single round of negative selection confirming the efficiency of our method (the only exception was the *ssrB* point mutant with 70% resolvants as shown in Fig. 1c).

## Discussion

Gene editing enables investigation of gene function. Here, we improved on a widely used method of consecutive single-crossovers. Our newly developed suicide vectors, based on a highly efficient dual negative selection strategy, mitigate the major pitfall of consecutive single-crossovers: the poor selection of resolvant clones after the second recombination. Thus, our new vectors do not require multiple rounds of counter-selection to ensure resolution of the suicide vector from the recipient strain. One of the two negative selection genes encodes I-SceI which recognizes a specific 18-basepair sequence [36]. While none of the strains used in this study harbored a I-SceI recognition sequence in their genomes, this sequence might be present in other bacteria which would need method adaptation. Gibson assembly enables rapid construction of plasmids with PCR fragments with no need for enzyme digestion and ligation, and no sequence constraints due to restriction sites. Our approach relies on endogenous RecA, but not the heterologous, powerful lambda-red recombinase, which might minimize the risk of secondary mutations. Purifying mutated loci by generalized phage transduction may thus not be required. Our method employs conjugation instead of electroporation (as required for lambda-red methods), which minimizes culture volumes and hands-on time. We anticipate that this method might be broadly applicable to additional bacterial species, including those for which recombineering has been difficult to implement.

## Conclusions

Our plasmids and protocols provide facile time-efficient methods for genetic engineering in multiple bacterial species including MDR clinical isolates.

## Methods

### Media and strains

Bacterial strains were cultured in Lennox lysogeny broth (LB) (tryptone 10 g/L, yeast extract 5 g/L and NaCl 5 g/L) medium. *E. coli* JKe201 [31] was cultured in the presence of 100 μM of diamino pimelic acid (DAP) (Sigma Aldrich D1377-10G). *Salmonella enterica* serovar Typhimurium SL1344 was cultured in LB in the presence of 90 μg/ml streptomycin (Sigma-Aldrich S9137-100G*). E. coli* EC01 [32] and *P. aeruginosa* UCBPP-PA14 were cultured in LB. For preparing chemically competent cells, fresh LB medium was inoculated at OD_600nm_ 0.01 with an overnight culture of JKe201 and grown until OD_600nm_ 0.4–0.6. Bacteria were washed twice with 25 ml of ice-cold 100 mM CaCl_2_ (Sigma Aldrich C1016-500G) solution containing 15% of glycerol (AppliChem, A1123,1000). Bacteria were resuspended in 5 ml ice-cold CaCl_2_ 100 mM / 15% glycerol and 200 μl aliquots were frozen and stored at − 80 °C. Super-Optimal broth with Catabolite repression (SOC) (tryptone 20 g/L, yeast extract 5 g/L, NaCl 0.5 g/L, KCl 0.186 g/L, MgSO_4_ 4.8 g/L and glucose 3.6 g/L) medium was used for resuspension after heat shock. 50 μg/ml kanamycin (Roth T832.4) or 15 μg/ml gentamicin (Gibco 15,750–037) were used to select *E. coli* transformants. For positive selection, kanamycin (Roth T832.4) at a final concentration of 50 μg/ml, gentamicin (Gibco 15,750–037) at a final concentration of 30 μg/ml, or potassium tellurite (Sigma P0677) at a final concentration of 10 μg/ml, were used. Counter-selection plates contained LB-no salt (10 g/L tryptone, 5 g/L yeast extract), 20% (w/v) sucrose (Sigma-Aldrich 84,097-1KG), 15 g/L agar and 0.5 μg/ml anhydrous tetracycline (AHT) (Sigma-Aldrich 37,919-100MG-R).

### Generation of the suicide vectors

Primers for generating pOPC-001, pOPC-003 and pFOK are reported in Supplementary Table S1. pOPC-001 was obtained by combining the kanamycin resistance cassette and the I-SceI restriction site from pWRG717 [37], the origin of replication (R6Kγ) and origin of transfer (oriT) from pGP704 [6, 38] and the *tetR* and *I-sceI* locus from pWRG730 [37] using Gibson assembly. pOPC-003 was generated by replacing the *tetR* and *I-sceI* locus from pOPC-001 with *sacB* from pEXG2 [39]. pFOK (5841 bp) was generated by inserting *sacB* amplified from pOPC-003 downstream of the *I-sceI* gene on pOPC-001. pFOG (5659 bp) was generated by replacing *aphA* of pFOK by *acc (3)-I*. pFOKT (6668 bp) was generated by insertion of *tpm* [35] between *aphA* and the multi cloning region (MCR).

### Amplification of the upstream and downstream regions

Flanking primers with a 40 bp overlap were designed to amplify 700 bp up- and downstream of the gene of interest using SnapGene® (version 4.0.3) with the Gibson Assembly tool (Supplementary Table S1). Fragments were amplified using a high-fidelity polymerase mix (KOD Hot Start Master Mix, Millipore) and separated on a 1% agarose gel. Vectors were purified from overnight cultures using a plasmid miniprep kit (ZymoPURE™, ZymoResearch). Vectors were digested using EcoRI-HF and BamHI-HF (New England BioLabs) for 1 h at 37 °C, or PCR-amplified, and purified on agarose gel. Alternatively, vectors can also be amplified by long-range PCR. Final concentrations of amplificated fragments and digested vectors were measured using a microvolume spectrometer (Colibri®).

### Gibson assembly and chemical transformation

Plasmids generated in this study are listed in Supplementary Table S2. Gibson assembly reaction was performed as described [27]. The reaction mix contained 50 ng of each up- and downstream fragments and 150 ng of suicide vector, and Gibson assembly mix 1x (New England BioLabs) in a total volume of 10 μl. The reaction mixture was incubated at 50 °C for 20 min. Five microliters of the reaction mixture was added to a 100 μl aliquot of *E. coli* JKe201 heat-shock competent bacteria and incubated for 30 min on ice. After a heat shock at 42 °C for 30 s followed by 2 min on ice, bacteria were resuspended in 1 ml prewarmed SOC medium containing 100 μM of DAP and incubated for 1 h at 37 °C. Transformants were selected on Lennox-LB agar plates containing 100 μM DAP (required for growth of JKe201) and either 50 μg/ml kanamycin or 15 μg/ml gentamicin. Clones were screened using PCR with primers oOPC-614 and oOPC-615 (Supplementary Table S1).

### Conjugation and selection of the first homologous recombination event

The recipient *S.* Typhimurium and *E. coli* strains were inoculated in 2 ml of LB containing no antibiotics at 37 °C. *P. aeruginosa* was inoculated in 2 ml LB without antibiotics at 42 °C. The donor *E. coli* strain was inoculated in 2 ml of LB containing 100 μM DAP but no antibiotics. Five hundred microliters each of overnight cultures of the donor *E. coli* strain and the recipient strain were washed with LB, mixed and centrifuged together. The pellet was resuspended in 50 μl LB and deposited on 22 mm filter membranes with 0.45 μm pores (Millipore, Merck) on a pre-dried LB agar plate. After mating for 6 h at 37 °C, bacteria were scraped from the filter membrane and resuspended in 1 ml LB. Merodiploid *S.* Typhimurium (pFOK) were selected on LB plates containing 90 μg/ml streptomycin and 50 μg/ml kanamycin at 37 °C for at least 16 h. *E. coli* (pFOKT) and *P. aeruginosa* (pFOG) merodiploids were selected on LB plates containing 10 μg/ml tellurite or 30 μg/ml gentamicin, respectively. Clones grew on tellurite to form black colonies.

### Counter-selection of the second homologous recombination event

At least three trans-conjugant colonies were combined and grown for 4 h at 37 °C in 2 ml of LB. Bacteria were then streaked on freshly prepared LB-no salt agar plates [24] containing 20% sucrose and 0.5 μg/ml AHT. Plates were incubated at 28 °C protected from light for at least 24 h. Colonies were screened for the desired mutation using PCR with external primers (Supplementary Table S1). Mutants were confirmed by DNA-sequencing (Microsynth.ch).

## Supplementary information

**Additional file 1: Figure S1.** Activity of the TetR system regulating expression of the green fluorescent protein (GFP) in absence and presence of its inducer anhydro-tetracycline (aTC) as measured by flow cytometry (AF, autofluorescence of a strain without *gfp*).

**Additional file 2: Figure S2.** Resolution results (mutant or reversion back to wild-type) for 50 colonies obtained after negative selection. The results for deletion of *foxA* were obtained from clone 5 shown in Fig. 1g.

**Additional file 3: Table S1.** Primers used in this study. **Table S2.** Plasmid used in this study.

## Data Availability

All data generated or analysed during this study are included in this published article.

## Abbreviations

AHT: Anhydro-tetracycline
CRISPR: Clustered regularly interspaced short palindromic repeats
DAP: Diaminopimelic acid
GFP: Green fluorescent protein
MDR: Multi-drug resistant
LB: Lysogeny broth
OD: Optical density
SOC: Super-Optimal broth with Catabolite repression
