# Supplementary figures and images for: Efficient dual-negative selection for bacterial genome editing
Source: BMC Microbiol. 2020 May 24;20:129. doi: 10.1186/s12866-020-01819-2 (PMC7245781; doi:10.1186/s12866-020-01819-2)

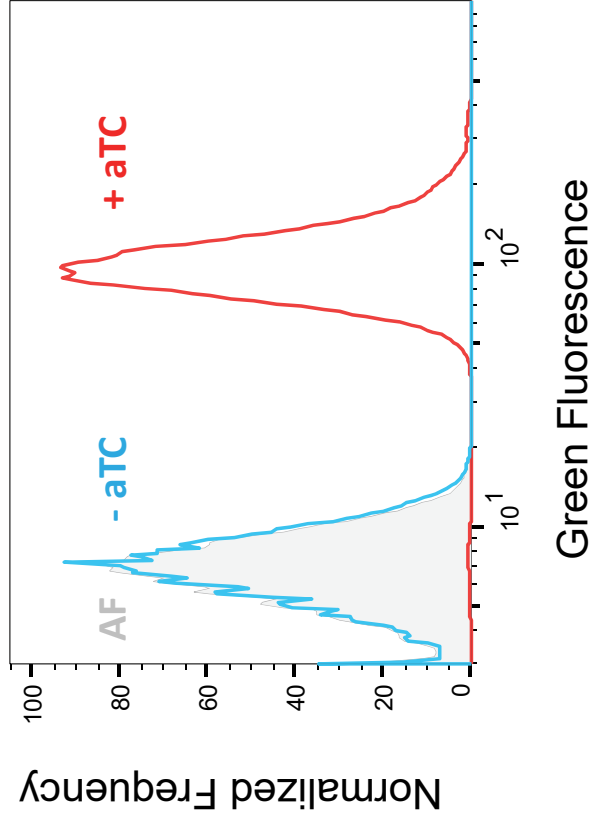

Supplement: Supplementary file 1 — Additional file 1: Figure S1. Activity of the TetR system regulating expression of the green fluorescent protein (GFP) in absence and presence of its inducer anhydro-tetracycline (aTC) as measured by flow cytometry (AF, autofluorescence of a strain without gfp). [file 12866_2020_1819_MOESM1_ESM.pdf]

Colonies

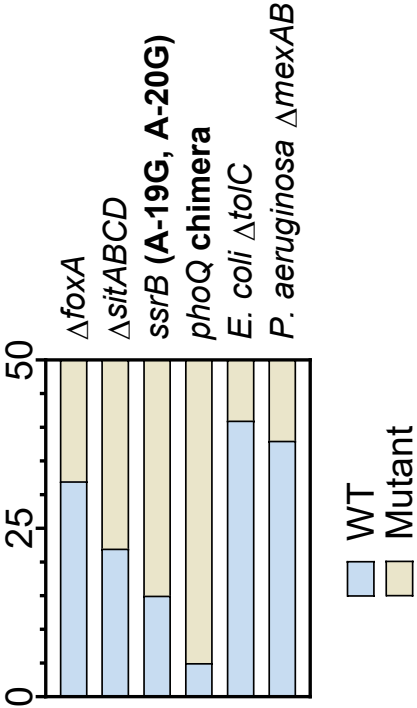

Supplement: Supplementary file 2 — Additional file 2: Figure S2. Resolution results (mutant or reversion back to wild-type) for 50 colonies obtained after negative selection. The results for deletion of foxA were obtained from clone 5 shown in Fig. 1g. [file 12866_2020_1819_MOESM2_ESM.pdf]
